# Emergency department use and responsiveness to the palliative care needs of patients with dementia at the end of life: A scoping review

**DOI:** 10.1017/S1478951524001627

**Published:** 2025-01-27

**Authors:** Sara Vieira Silva, Paulo Conceição, Bárbara Antunes, Carla Teixeira

**Affiliations:** 1 Palliative Care Service, Unidade Local de Saúde de Santo António, Porto, Portugal; 2 Internal Medicine Service, Unidade Local de Saúde de Santo António, Porto, Portugal; 3 Department of Public Health and Primary Care, University of Cambridge, Cambridge, UK; 4 Unidade Local de Saúde de Santo António, ICBAS Universidade do Porto, Porto, Portugal

**Keywords:** Dementia, end-of-life care, emergency medical services, palliative care, palliative care needs

## Abstract

**Objectives:**

More than 50% of patients with dementia visit the emergency department (ED) each year. Patients with dementia experience frequently unrelieved symptoms that can benefit from *palliative care*. Response to *palliative care* needs in the ED can be quite challenging and access to *palliative care* is generally scarce. The aim of this scoping review is to assess ED use and responsiveness to *palliative care* needs of patients with dementia in their last year of life.

**Methods:**

A scoping literature review following the Joanna Briggs Institute methodology. Electronic search of the literature was undertaken in Medline (PubMed), Web of Science, Scopus, Scielo, and APA PsycInfo, last updated on 19 February 2024.

**Results:**

Twenty-four studies were identified and confirmed that patients with dementia frequently resort to the ED near the end of life, frequently more than once in their last year of life. Eight studies directly addressed *palliative care* needs, suggesting significant rates of *palliative care* needs among patients with dementia and in comparison, to other oncological or non-oncological conditions. Infections and neuropsychiatric symptoms were the main reasons of admission to the ED. Access to *palliative care* was confirmed to be low.

**Significance of results:**

This scoping review indicates that patients with dementia frequently resource to the ED in their last year of life with unmet *palliative care* needs. Although scarce access to *palliative care* and the existence of important barriers in the ED, *palliative care* intervention in this setting can be seen as an opportunity to attend *palliative care* needs and referral to *palliative care* services.

## Introduction

Emergency departments (EDs) are generally designed to respond to acute and life-threatening conditions, so visits to the ED by people who are approaching end of life frequently can be distressing for patients, families and challenging for staff (Antunes 2020; Briggs et al. 2013). Up to more than 50% of patients with dementia visit the ED each year (Hunt et al. 2018a) and as the population over 65 years old continues to grow, estimates suggest that from 57.4 million cases worldwide in 2019 this number will increase up to 152.8 million cases in 2050 (Nichols et al. 2022) and consequently increase ED use. Patients with dementia experience frequently unrelieved pain (Reynolds et al. 2008) and other significant needs (Black et al. 2006; Hendriks et al. 2014; Kverno et al. 2008; Shega et al. 2006) that can benefit from palliative care interventions. A 2021 Cochrane review (Walsh et al. 2021) showed that advance care planning interventions for people with advanced dementia increased the documentation of advance directives and the number of discussions regarding goals of care with family decision-makers and may increase concordance with goals of care. Although not with the same grade of evidence, previous studies have identified other potential benefits of palliative care such as improvement of symptom burden, prevention of under or overtreatment, and enhance caregiver quality of life (Mitchell et al. 2009; van Soest-poortvliet et al. 2015). Also, community-based palliative care has been associated with a reduction of ED visits (Godard-Sebillotte et al. 2019; Williamson et al. 2021), resulting in lower health-care costs and greater satisfaction with care among patients and families.

In general, at the end of life, several studies have shown poor quality of care for patients with dementia, including low levels of symptom control (Davies et al. 2014; Martinsson et al. 2018). The ED reality includes rapid decision-making, aggressive disease-modifying therapy (Lafond et al. 2016), long waiting times, lack of communication (Smith et al. 2010), bright lights, and constant noise, all of which can constraint the care provided, specially to vulnerable populations.

In a recent systematic review, Williamson et al. (2021) identified significant factors that are associated with ED attendance to patients with dementia at the end of life, namely, number of comorbidities, neuropsychiatric symptoms, previous hospital transfers, and rural living. These were positively associated, while higher socioeconomic status, being unmarried and living in a care home were negatively associated, among other factors that were also identified. Although these factors might constitute relevant criteria to help identify patients at risk of ED attendance, there is a lack of knowledge about ED assistance to patients with dementia with palliative care needs at the end of life. This scoping review aimed to assess ED use and responsiveness to palliative care needs of these patients in their last year of life, focusing mainly on ED resource, the opportunities, and barriers to palliative care.

## Methods

This scoping review follows the Joanna Briggs Institute methodology recommendations for scoping reviews (Peters 2015) and the Preferred Reporting Items for Systematic Reviews and Meta-analysis Protocol (PRISMA-P) for Scoping Reviews protocol (Tricco et al. 2018). The final protocol is available from the corresponding author upon request. The PRISMA for Scoping Reviews checklist is attached in Appendix 1.

### Selection criteria

Studies addressing palliative care needs of patients with dementia who attended the ED were considered. To be eligible, studies had to include patients with dementia at the end of life, defined as been presumably in the last 12 months of life (Fisher et al. 2015). All types of dementia were considered. Intellectual disability was not considered. Studies did not need to present a control group. Peer-reviewed studies – either quantitative, qualitative, or mixed-methods – were eligible. Editorials, letters to the editor, and case studies were ineligible. Only studies written in English, Portuguese, or Spanish were included.

Only studies authorized by an ethics committee, respecting the principles of Helsinki Declaration, were considered.

### Search strategy

The search strategy was defined using PRISMA-P (Tricco et al. 2018) which was revised by the research team. Studies were retrieved from Medline (PubMed), Web of Science, Scopus, Scielo, and APA PsycInfo using keywords and controlled vocabulary representing the terms “Palliative Care needs,” “Patients with dementia,” and “ED,” with adjustment to the different databases (Table 1). As an example, detailed search strategy for Medline is available in Supplementary Table S1. Gray literature was considered and consisted of searching Google Scholar and abstracts from health conferences. Hand search was conducted based on bibliographies from previous reviews. The search was last updated on 19 February 2024, and final search results were exported to EndNote, where duplicates were removed.

**Table 1.** Search terms used for scoping literature review

**Population** “Dementia” OR “Alzheimer’s Disease” OR “Dementia Multi Infarct” OR “Vascular Dementia” OR “Frontotemporal Dementia” OR “Lewy Body Dementia” OR “Korsakoff’s Dementia” OR “HIV Dementia” OR “Creutzfeldt-Jakob’s Dementia” OR “Cognitive Impairment” OR “Senile” terms AND **Intervention** “Emergency Department” OR “Emergency Care” OR “Emergency Service” OR “Emergency room” OR “Emergency Medicine” OR “Acute Care” terms AND **Outcomes** “Palliative Care” OR “Palliative Medicine” OR “Terminal care” OR “Dying” OR “End of life Care” OR “Supportive care” OR “Hospice care” terms

### Screening and data extraction

Two independent reviewers (S.S. and P.C.) performed a preliminary screening of titles and abstracts. Eligible articles were identified, and full-text reading was performed by the same reviewers who further excluded articles if they did not meet the inclusion criteria. Discrepancies were resolved by discussion and consensus.

According to Joanna Briggs Institute methodology recommendations for scoping reviews, data were collected, collated, and summarized (Peters 2015). A data extraction form was developed by the research team, available in Appendix 2, and included main characteristics of the studies (identification of first author, date of publication, country, objective, design, population, sample size, recruitment, date of data collection, interventions, comparators, outcomes, and statistical analysis), the prevalence of patients with dementia at the end of life who presented palliative care needs, the reasons for resorting to the ED, the benefits, barriers, and lack of responsiveness to these needs. The 2 independent reviewers performed the data extraction with subsequent joint discussion and validation of the collected data by a third independent reviewer.

### Analysis, synthesis, and reporting

A content analysis of the articles was conducted following the 7-step approach (Faria-Schützer et al. 2021): 1. Editing material, 2. Free-floating readings, 3. Construction of the units of analysis, 4. Identification of core of meanings, 5. Consolidation of categories, 6. Discussion of topics, and 7. Validity. Content is presented in tables with summarized items and with a complementary narrative format. Critical appraisal of the included studies was assessed according to Gough (2007) Weight of Evidence (WoE) (Gough 2007), a tool that measures overall quality as low, medium, or high.

## Results

**Figure 1.**
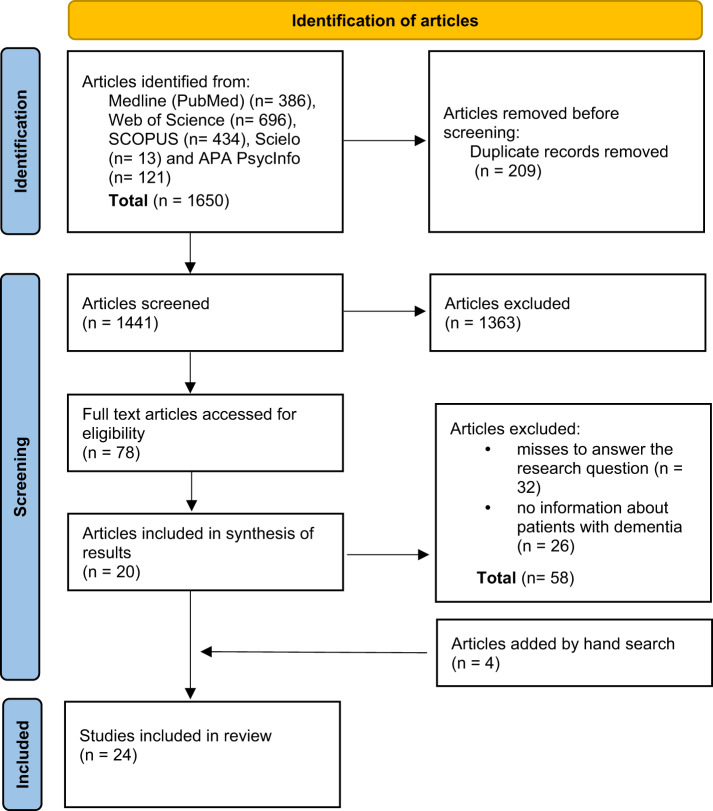
PRISMA flow-diagram of selection of source of evidence.

From 1441 initial records, 78 papers were screened for eligibility and 24 were included in the full review (Fig. 1).

An overview of the characteristics of the 24 included studies is provided in Table 2. Only 2 studies took place in developing countries (Amado-Tineo et al. 2020a, 2020b), the majority occurred in Europe (Afonso-Argilés et al. 2020; Antunes et al. 2021; De Matteis et al. 2022; Fürst et al. 2022; Leniz et al. 2021; Nordt et al. 2023; Reeves et al. 2023; Sleeman et al. 2018; Willert et al. 2021; Williamson et al. 2023) or North America (Glajchen et al. 2011; Hanson et al. 2019; Hill et al. 2022; Hunt et al. 2018b; Kirkland et al. 2022a; Kruhlak et al. 2021; Lamantia et al. 2016; Shah et al. 2022). Quantitative methods were used for all of the studies (*n* = 24), 2 were randomized controlled trials (Hanson et al. 2019; Shah et al. 2022); from the remaining, 6 of the observational were prospective studies (Afonso-Argilés et al. 2020; Amado-Tineo et al. 2020a; Kirkland et al. 2022a; Kruhlak et al. 2021; Sleeman et al. 2018; Straeuli et al. 2022). Kirkland’s study (Kirkland et al. 2022a) is a secondary analysis of the Kruhlak (Kruhlak et al. 2021). Most of the studies were conducted in more than 1 centre (Afonso-Argilés et al. 2020; Amado-Tineo et al. 2020a; Chen et al. 2017; Fürst et al. 2022; Hill et al. 2022; Hunt et al. 2018b; Kirkland et al. 2022a; Kruhlak et al. 2021; Leniz et al. 2021; Reeves et al. 2023; Rosenwax et al. 2015; Shah et al. 2022; Sleeman et al. 2018; Williamson et al. 2023; Wong et al 2017). Sample sizes ranged from 7 patients with dementia (Amado-Tineo et al. 2020a; Willert et al. 2021) to 918,341 patients (Reeves et al. 2023).

**Table 2.** Main characteristics of the 24 included studies

Author, year of publication	Study design/setting/(country)	Sample size/patients with dementia	Aim	Prevalence of patients with dementia with PC needs	PC assessment tool	Other main findings	Quality WoE D
1. Glajchen M36, 2011	Prospective, intervention study ED 1 university hospital (USA)	139/36	Address the needs of elderly patients presenting to ED and explore the feasibility of rapid screening and referral	7.9% (18.3% of patients with dementia attended at ED)	BriefPal	>> Primary diagnosis of patients with PC needs: cancer 43%, dementia 22%, and heart failure 16% >> Pain was the most common symptom – 14%, dyspnea – 12%, nausea/diarrhea/anorexia/weight loss – 8% >> Caregivers of patients in the referral group tended to show significantly higher levels of burden >> Patients that received PC reported symptom reduction, 80% died – 25 in hospital, 8 at hospice, 5 at home, and 3 at nursing home >> Rate of referral to PC and hospice rise during the implementation phase, with referral peaks at the midpoint, after staff education and during the months when the project social worker was in the ED full time, with a marked decline when she was away >> Main barrier to referral was reluctance by the primary care physician, included fear of involving PC, misunderstanding PC and hospice, and belief that they were already “doing” PC. >> System barriers included lack of understanding about PC and hospice and hesitation from ED+ staff about involving another medical specialty. The PC service itself was in a state of flux, with a gap in attendings for the duration of the project. >> Patient and family barriers included uncertainty about the patient’s prognosis and the symbolic meaning of PC and hospice >> The BriefPal project showed the feasibility of successful screening, the value of staff education, and the importance of trained champions	Medium
2. Rosenwax45, 2015	Retrospective, observational study Data from WA Linkage System (ED of Western Australian state) (Australia)	8126/5261	Describe patterns in the use of ED by patients with dementia in their last year of life and determine whether this is modified by the use of community-based PC service	Not directly evaluated	-	>> 6% of the dementia cohort used community-based palliative care in the last year of life compared to 26% of the comparative cohort >> >70% of decedents in both the dementia and comparative cohorts attended an ED at least once in the last year of life >> Visits to the ED by the dementia cohort tended toward being triaged as less urgent >> The dementia cohort had a higher proportion of neurological and mental disorder presenting symptoms and a higher proportion of fall- and injury-related symptoms at presentation to ED. In contrast, the comparative cohort had more cardiac and abdominal pain-related presentations to ED >> At the time of death, decedents with dementia in other disease attended ED on average of 2.7 (95% confidence interval (CI): 2.4–3.0) days compared to 2.7 (95% CI: 2.6–2.8) days for the comparative cohort. >> For the first 130 days of the last year of life, those receiving regular care in a private residence visited ED almost twice as often as those receiving palliative care (hazard ratio 1.9; 95% CI: 1.4–2.5). Decedents with dementia who received regular care in a care facility visited the ED 1.4 times more often than those receiving community-based palliative care (95% CI: 1.1–1.9). In the last month of life, the relative rate of ED visits for those receiving regular care jumped markedly compared to those receiving palliative care reaching a maximum rate around day 340 or 25 days before death. Those receiving regular care in private residences visited EDs 6.7 (95% CI: 4.7–9.6) times more frequently and those receiving regular care in a care facility visited EDs 3.1 (95% CI: 2.2–4.2) times more frequently than those of dementia cohort who were receiving palliative care at that time. >> Other factors associated with increased ED use in the dementia cohort were being male, being younger and living with dementia with other diseases rather than Alzheimer’s or vascular dementia, living in outer regional and remote areas, and having certain types of comorbid conditions	High
3. LaMantia 37, 2016	Retrospective, observational study ED of 1 university hospital and database from 10 federally qualified health centers (USA)	32697/11069	Describe the clinical characteristics, care patterns, associated medical costs, and health outcomes for older adults with and without dementia who seek care in the ED	Not directly evaluated	-	>> 37–54% of patients with dementia visited ED/y vs 20–31%/y of patients without dementia >> Patients with dementia were admitted to the hospital at higher rates (39.7%) than patients without dementia (29.06%) (*p* < 0.001) >> 58% of patients with dementia had at least 1 ED visit within 30 days after an index ED visit as compared to 38% of patients without dementia (*p* < 0.001) >> Mean of comorbid conditions of patients with dementia were 8.0 vs without 5.0 (*p* < 0.001) >> Most frequent ED primary diagnosis: urinary tract infections, chest pain and abdominal pain; those not discharged, pneumonia, congestive heart failure, and urinary tract infections >> Mortality of dementia patients at >ED 1.3% vs 1.0% >> Mean Medicaid payment was increased ($199 vs $134, *p* < 0.001) for patients with dementia as compared to those without dementia. Mean Medicare ED payments for patients with dementia at any time during the study period were 75% higher for patients without dementia ($6028 vs $3454, *p* < 0.001)	Medium
4. Wong J46, 2017	Retrospective, observational study ED of 2 general hospitals (New Zealand)	1024/50	Estimate the incidence of patients presenting to ED with PC needs	1.2% (24.0% of patients with dementia attended at the ED)	GSF PIG	>> Cancer, chronic obstructive pulmonary disease, and heart failure were the most common disease presentations (75.1%), dementia (4.9%) >> Most common symptoms: pain (23.4%), dyspnea (19.1%), and “unwell” (16.0%) (Fig. 5). >> 18.4% of the overall patients presented PC unmet needs vs 24.0% patients with dementia	Medium
5. Sleeman KE26, 2018	Retrospective, observational study SLAM Biomedical Research Centre Case Register (United Kingdom)	4867/4867	Examine the frequency, and identify the predictors, of ED attendance among people with dementia in their last year of life	Not directly evaluated	-	>> Factors associated with increased number of ED attendances during the last year of life: male gender, depression severity, higher cognitive function, and diagnosis of vascular dementia. >> Being resident in a care home and living in the most affluent areas were associated with fewer ED attendances	Medium
6. Chen YH47, 2017	Retrospective, observational study Data from the Taiwan National Health Insurance Research Database (Taiwan)	2724/908	Comparison of the utilization of health-care services and life-sustaining interventions by patients with dementia and cancer patients in their last year before death.	Not directly evaluated		>> Number of patients with dementia with <3 ED visits/year 287 (62.9%) vs with cancer 613 (62.7%). Number of patients with dementia with ≥3 ED visits/year 169 (37.1%) vs with cancer 364 (37.3%) >> Most common causes of hospitalization and ED visits by dementia and cancer groups were infectious diseases	High
7. Hanson LC37, 2019	Randomized controlled trial ED of 1 university	62/62	Develop a best-practice model of specialty PC for late-stage dementia, and conduct a pilot randomized trial of specialty PC for late-stage dementia triggered by	Not directly evaluated	-	>> The primary outcome of 60-day hospital or emergency department visits did not differ significantly between intervention and control arms (0.68 vs 0.53 visits, *p* = 0.415) >> The most common admitting diagnoses were infections and neuropsychiatric symptoms >> Two-thirds of patients had advance directives. Baseline characteristics did not differ significantly between	High
	hospital (USA)		hospitalization for acute illness			study arms >>Family decision-makers had an average age of 59.7 years, 79% were female, and more than half were	
						daughters. At enrollment, 92% of caregivers felt they were very involved in treatment decisions for the person	
						with dementia, and 60% expected that person to get worse or possibly die in the next 6 months	
						>> One of 3 enrolled patients with late-stage dementia visited an emergency department or was hospitalized	
						in the 60 days after discharge	
						>> Family caregivers’ ratings of comfort for the person with dementia and of distress for themselves did not	
						differ between arms at 60 days	
						>> Dementia patients in the intervention arm had more elements of clinical palliative care addressed in their	
						hospital treatment plan, as measured on the 10-point Palliative Care Domain score (7.6 vs 2.7, *p* < 0.001)	
						>> Patients with the intervention group were more likely to have assessment and treatment for physical	
						symptoms of dyspnea, constipation, and nausea, and for neuropsyquiatric symptoms.	
						>> Spiritual needs were addressed for 47% of patient–family dyads in the intervention arm, and for 0% of those	
						in the control arm	
						>> Specialty PC during hospitalization also resulted in increased communication and decision-making about	
						treatments relevant to late-stage dementia and in increased hospice use	
8. Hunt LJ38, 2018b	Retrospective, observational study Data from The	281/281	Assess prevalence of pain and unmet need for pain management in the last month of life in older adults dying with dementia; describe ED use in the last month of life	Not directly evaluated	-	>>The most frequent diagnoses for ED visit were septicemia; cardiac arrest and pneumonia and other respiratory disease. >> Almost 3/4 of respondents reported that the decedent had pain in the last month of life (73.1%) and approximately 10% had an unmet need for pain management	Medium
	National Health and Aging Trends Study (USA)		among older adults with dementia; and examine whether pain was associated with increased ED use in the last month of life in older adults with dementia			>> More than half (56.6%) of decedents with dementia visited the ED in the last month of life. The mean number of ED visits was 0.75. >> There was no association between pain in the last month of life and ED visits. However, unmet need for pain management increased ED visit rate by almost 50%. A higher percentage of participants with unmet need for	
						pain management had 1 (46% vs 39%) or multiple ED visits (26% v.s 16%) compared to those without	
						unmet need for pain management	
9. Afonso-Argilés FJ 26, 2020	Prospective, cross-sectional observational study Home Care and Nursing Care (Estonia, Finland, France, Germany, Netherlands, Spain, Sweden, United Kingdom)	1700/284	Assess frequency of hospital admissions (including ED), among people with dementia from 8 European countries living in the community and those living in nursing homes; examine the factors associated with hospital admissions among people with dementia living in the community and those living in nursing homes settings, separately; and to evaluate the costs associated with hospital admissions according to the different living situations	Not directly evaluated	-	>> 16.7% patients with dementia had ED visit >> Estimated rate of ED per person with dementia/year of 0.26 among persons living in nursing home and 0.43 among those living in home care >> Factors associated with ED visit: people with dementia living in nursing home polypharmacy (OR 1.96, CI (1.03–3.75); people with dementia living in home care, presence of falls in the preceding 3 months (OR 1.64, CI (1.12–2.41)), polypharmacy (OR 2.48, CI (1.70–3.62)), weight loss (OR 1.73, CI (1.17–2.56)), caregiver burden (OR 1.76, CI (1.04–2.98)). >>The estimated average expenditure per person with dementia/year for ED admission was 67.30€ in the nursing home group and 117.48€ in the home care group	High
10. Amado-Tineo JA23, 2020a	Prospective, cross-sectional, observational study ED of 3 tertiary hospitals (Peru)	60/7	Estimate the prevalence of advance disease among patients admitted to ED and identify among them the need of PC in patients admitted to ED	83.3% of the overall patients (not specifically patients with dementia)	NECPAL	>> Surprised question:”no” answer in *n*=26 (43.3%) of overall patients >> ED professional considered that the patient needed PC in *n* = 50 (83.3%), requested PC for the patient in *n* = 12 (20.0%), the patient used PC in *n* = 13 (21.6%) >> Symptoms were controlled in *n* = 8 (13.3%) of patients >> Reason for admission was uncontrolled symptoms in 52 (86.7%) patients, with the most frequent being dyspnea (42%), somnolence (22%), and pain (18%)	Medium
11. Amado-Tineo JA25, 2020b	Retrospective, observational study ED of 1 university hospital (Peru)	233/36	Identify the frequency and characteristics of patients with chronic terminal disease admitted to the ED of a national hospital and identify the reason for admission, treatment received and destination, comparing oncological and non-oncological pathology	4.8% of the overall patients (not specificaly patients with dementia)	SPICT-ESTM	>> ED resource cause: non--oncological – infection 80 (59.7%), pain 3 (2.2%), encephalopathy 17 (12.7%), bleeding 7 (5.2%), dyspnea 8 (6.0%) vs cancer infection 31 (31.3%), pain 17 (17.2%), encephalopathy 2 (2.0%), bleeding 10 (10.1%), dyspnea 7 (7.1%) >> Invasive procedures: non-oncological – nasogastric tube 66 (49.3%), orotraqueal tube 15 (11.2%), vasopressors 7 (5.2%), invasive ventilation 12 (9.0%), hemodialysis 4 (3.0%), blood transfusion 9 (6.7%) vs cancer nasogastric tube 13 (13.1%), orotraqueal tube 4 (4.0%), vasopressors 11 (11.1%), invasive ventilation 3 (3.0%), hemodialysis 1 (1.0%), blood transfusion 15 (15.2%) >>Treatment: Non–oncological – a ntibiotics 80 (59.7%), opioids 2 (1.5%), metamizol 11 (8.2%), haloperidol 10 (7.5%) vs antibiotics 31 (31.3%), opioids 10 (10.1%), metamizol 5 (5.1%), haloperidol 1 (1.0%) >> 27% ED mortality of 234 patients with PC criteria (not specifically dementia patients).	Medium
12. Antunes A28, 2021	Retrospective, observational study ED of 1 general hospital (Portugal)	484/45	Examine the influence of chronic diseases in ED and inpatient hospital utilization and expenditures in the 12 months before death	Not directly evaluated	-	>>Median of 3 EDadmissions/year for patients with dementia vs 2 in overall patients during the last year of life	High
13. Kruhlak M40, 2021	Prospective, cross-sectional observational study ED of 2 hospitals, 1 tertiary (Canada)	664/151	Identify presentations for end of life conditions to ED and describe the characteristics of the patients and their management	2.5% of the overall patients, 78.0% of patients at the end of life (neither specifically patients with dementia)	P-cares	>> Most common EOL condition cancer (41%), followed by dementia (23%) >> 78% of the presentations for EOL conditions met the criteria for unmet PC needs. Most common risk factors: a positive response to the SQ (68%), followed by uncontrolled symptoms (55%), and frequent ED visits/hospitalizations in the previous 6 months (54%). >> Goals of care were documented in two-thirds of patients 62% (396) and considered appropriated by physicians in 59% (235) >> Physicians reported challenges in providing care for 24% of the presentations, most commonly due to a language barrier (34%) or cognitive impairment (34%) >> Reason for admission: Dyspnea 21% (121), general weakness 12% (76), abdominal pain 9% (59), altered level consciousness (4%, 28) >> 84% of presentations involved medical treatment, with antibiotics being the most common medication prescribed (35%), followed by opioids (34%). Almost all the presentations involved at least1 investigation during their ED visit (97%) with blood work (97%), imaging (92%), and electrocardiogram (58%) being the most common investigations. An estimated 11% of presentations involved various medical procedures, with noninvasive positive pressure ventilation (43%) and intubation (30%) being the most common >> 0.9% of presentations included admission to a PC unit. Nearly 30% of presentations included a postdischarge referral, which included home care (32%), specialists (30%), and palliative services (26%). >> A total of 16% of the patients died during their index presentation either in the ED or in hospital, most of whom presented with advanced cancer (48%), dementia (19%), or chronic pulmonary disease (16%) >> Patients with dementia (OR: 0.32; 95% CI:0.22–0.48) were less likely to have presented to the ED in the previous 6 months	Medium
14. Leniz J29, 2021	Retrospective, observational study. Data from the Discover dataset (United Kingdom)	5804/5804	Explore the proportion and characteristics of people with a diagnosis of dementia who are identified as having PC needs during the last year of life; examine the association between identification of PC needs before the last 90 days and multiple non-elective hospital admissions, primary and community care contacts during the last 90 days of life	33.6%	Palliative Care Quality and Outcomes Framework	>> 2576 (44.4%) with 3 or more comorbidities, hypertension was the most common 3742 (64.5%) >> 12.7% (737) experienced multiple non-elective hospital admission in the last 90 days of life >> 13.7% (639) dementia patients without PC identification had multiple hospital admissions in the last 90 days of life VS 8.6% (98) from those who had PC identification (OR 0.70) >> Among the 5804 decedents with dementia, 1132 (19.5%) and 2658 (45.8%) had at least 1 face-to-face or telephone contact with the primary care practice in the last 90 days of life. 2782 (47.9%) people in the cohort had at least 1 contact with community nurses in the last 90 days >> A total of 403 (6.9%) patients had at least 1 contact with community PC teams, 589 (10.1%) with physiotherapists and 638 (11.0%) with rehabilitation teams	Medium
15. Willert AC30, 2021	Retrospective, observational study ED of 1 university hospital (Germany)	130/7	Identification of causes for ED hospitalization of patients with neurological disorders or neurological symptoms requiring PC	All patients included had PC needs	Not specified	>> Primary diagnoses: neoplastic disorders (49%), neurodegenerative disorders (30%) (dementia [5.4%]), cerebrovascular diseases (18%) >> Reasons for admission: epileptic seizures (22%), gait disorder/falls (22%), disturbances of consciousness (20%), pain (17%), nutritional problems (17%), and paresis (14%), difficulties with organization of care or overburdening of family (8%) >> Symptom assessment: assistance with Activities of Daily Living (ADL, 83%), weakness (71%), difficulties with organization of care (61%), tiredness (59%), or overburdening of family caregivers (53%), pain (31%), difficulties in communication (30% aphasia, 38% dysarthria), nutritional problems for solids (42%) or fluids (38%), and paresis (47%). >> Diagnostic and therapy: at least 1 mode of acute diagnostic imaging (88%), cranial imaging was most frequently performed (60%); in 55% of cases, a medication was administered in the ED, antiseizure medication (19%) and benzodiazepines (14%), analgesics (26%), antibiotics (17%) >> Documentation of Healthcare Proxy or Legal Guardian, Patient Decree, and Therapy Limitations in 31%. >> In 63% of patients, it took at least 2 days until specialist PC consultation was initiated >> Forty percent of patients were discharged with further inpatient (PC unit or hospice; 12%) or outpatient (home/nursery home with outpatient PC supply; 28%) specialized PC	Medium
16. Furst 31, 2022	Retrospective, observational study Data from the Stockholm Region’s central data warehouse (Sweden)	12667/605	Compare 2 groups of > 65 years old deceased patients with cancer and patients with concomitant cancer and dementia at end of life regarding their access to specialized PC; study their ED visits, hospital admissions, and place of death	Not directly evaluated	-	>> Concomitant dementia diagnosis with cancer did not affect the likelihood of ED visits (259 (43%) vs 4768 (40%) *p* value 0 .11) >> During the last 3 months of life, 76% of patients with cancer and 42% of patients with cancer and concomitant dementia had access to specialist PC (*p* <.0001) >> Specialist PC for patients with concomitant cancer and dementia did not influence ED visits during the last month of life but affected place of death: 3% and 21%, among those with and without access to PC, respectively of hospital deaths (*p* < 0.001)	High
17. Hill JD41, 2022	Retrospective, observational study Data from Medicare claims referring to 34 ED (USA)	29626/29626	Identify a large sample of community-dwelling people living with dementia from a diverse cohort of EDs and compare post-ED outcomes to patients with chronic conditions without dementia	Not directly evaluated	-	>> Patients with dementia had a higher percentage of ED visits (55.2% vs. 43.8%). >> Patients with dementia had a greater number of chronic conditions (mean 4.8+- 2.8 vs 3.3, SD: 2.1) >> Disposition to home was lower for patients with dementia (37.9% vs 53.6%) and to hospice slightly higher (0.4% vs. 0.2%). >> Patients with dementia had higher health-care utilization, including 1 or more ED visits post-index visit (43.1% vs 35.4%), greater number of inpatient stays post-index visit (mean 0.8 visits vs 0.5 visits) and ED visits post index (mean 0.9 visits vs. 0.7 visits), 1 or more inpatient stays post-index visit (43.4% of patients vs 28.0%), and longer length of inpatient stays (mean 6.5 days vs 5.9). >> Patients with dementia who died within the 12 months following their index visit were nearly double (31.9% vs 15.3%)	High
18. Kirkland S42a, 2022a	Prospective, cross-sectional observational study ED of 2 hospitals, 1 university hospital (Canada)	663/106	Identify and compare the characteristics and management of ED presentations for advanced cancer to other end of life conditions	76.5% of patients with non-cancer conditions that included patients with dementia	P-cares	>> No diference between presentations by patients with unmet PC needs, 81% cancer vs 77% other conditions. Emergency physicians were more likely to report a positive response to the surprise question for patients with cancer (76% vs 63%) >> Goals of care documentation was lower among patients with cancer (53% vs 75%) >> Patients with cancer were more likely to be referred to PC services (41% vs 10%, *p* < 0.001) at discharge. >> PC consultations in the ED were requested in only 7 (1%) presentations, all for patients with cancer. >> There were no significant differences in proportion of patients with a return to ED within 30 days or a previous ED visit 6 months before	High
19. Shah MN43, 2022	Randomized controlled trial ED of 3 university hospitals (USA)	81/81	Evaluate if care transition intervention improves ED to home transitions for cognitively impaired community-dwelling older adults	Not directly evaluated	-	>> No significant difference in the primary outcome of ED revisits within 30 days. Among secondary outcomes, no significant differences in ED revisits within 14 days or outpatient follow-up at 14 or 30 days >> Multivariate regression analyses for ED revisits, adjusted for presence of moderate-to-severe depression and health literacy, showed that intervention participants had 75% decrease odds of an ED revisit within 30 days (OR 0.25, 95% CI 0.07–0.90) >> Forty-three of the 80 participants had specified red flags on their ED discharge instructions. Seven (16.3%) of these participants correctly reported at least 1 specific red flag from their ED discharge instructions, with no significant difference between groups. >> Specific medication changes were present in 21 participants. >> ED patients randomized to the intervention group had a 26% increase odds of obtaining follow-up.	High
20. Straeuli C44, 2022	Prospective, cross-sectional observational study ED of 1 general hospital (South Africa)	426/62	Estimate the prevalence of patients with potential PC needs attending the ED; describe the patient demographics and diagnoses of these patients; to understand the reasons for ED attendance and describe their disposition from the ED	0.6%	Palliative Care Identification tool-based GSF-PIG	>> 4.24% (426) of the total patient visits to the ED (N = 10 049) presented PC needs >>The top 3 diagnoses were cancer (25.8%), neurological disease (19.7%) and HIV (17.4%) >> Reasons for ED admission: physical symptoms (*n* = 370 (87%)), of which pain and dyspnea were most frequently reported, followed by social (46 (11%)) and health systems (43 (10%) ) needs >> Most of the patients with social reasons for attending had caregiver fatigue or absent support structures, needing placement. Health system reasons included catheter changes or running out of medication at home	Medium
21. De Matteis G32, 2022	Retrospective, observational study ED of 1 university hospital (Italy)	273559/4896	Describe the clinical characteristics of older patients affected by dementia and hospitalized, investigate the impact of dementia on in-hospital mortality, and identify the predictors of in-hospital mortality in these patients	Not directly evaluated	-	>> Reasons for ED admission (patients without dementia vs with dementia): dyspnea 9096 (20.0) vs 729 (20.5); Fever 8520 (18.8) vs 782 (22.0), chest pain 5817 (12.8) vs 161 (4.5), syncope 3420 (7.5) vs 355 (10.0), abdominal pain 5324 (11.7) vs 207 (5.8), diarrhea 1553 (3.4) vs 134 (3.8), vomit 3752 (8.3) vs 256 (7.2), malaise/fatigue 5411 (11.9) vs 374 (10.5) >> Acute medical conditions (patients without dementia vs with dementia): respiratory failure 5904 (13.0) vs 681 (19.1), COPD exacerbation 1651 (3.6) vs 147, acute heart failure 5172 (11.4) vs 619 (17.4), acute myocardial infarction 2798 (6.2) vs 83 (2.3), acute atrial fibrillation 6741 (14.8) vs 569 (16.0), pulmonary embolism 702 (1.5) vs 51 (1.4), ischemic stroke 4549 (10.0) vs 482 (13.5), hemorrhagic stroke 1947 (4.3) vs 225 (6.3) <0.001, bloodstream infections 3084 (6.8) vs 372 (10.5), pneumonia 4386 (9.7) vs 733 (20.6), abdominal infection 1743 (3.8) vs 102 (2.9), urinary tract infection 3729 (7.2) vs 477 (13.4) <0.001, acute kidney injury 2961 (6.5) vs 376 (10.6) >> Death during hospitalization (patients without dementia vs with dementia): 4865 (10.7) vs 666 (18.7)	High
22. Nordt SP33,^2023^	Retrospective, observational study ED of 1 university hospital (Ireland)	499/29	Identify and assess the frequency of presenting complaints, diagnosis, triage acuity, and admission, in ED patients previously evaluated by a university-hospital-based palliative medicine service	Not directly evaluated	-	>> 499 ED visits, of which, 92 (18.44%) patients being seen more than once in the ED >> 29 (5.81%) patients with dementia, second non-oncological most common cause. 3.1 mean number of repeat ED visits of these patients, median of 3, and a range of 2–9 visits >> 358 (71.7%) patients admitted and 141 (28.3%) discharged home >> 373 visits on weekdays (74.75%) compared to 126 visits on weekends (25.25%), as weekends account for 28% of a week >> 42% presented low emergency scores >> 64 (12.8%) admitted patients died during their hospital admission	Medium
23. Reeves D34,^2023^	Retrospective, observational study ED of 178 general hospitals (United Kingdom)	2569007/918341	Describe the epidemiology of ED admissions for patients with dementia and how this compares to people without across the period 2010–2016; examine the trends in hospital outcomes for patients with dementia and without across the period and the extent to which differences in outcomes might be accounted for by differences in patient characteristics	Not directly evaluated	-	>> Patients with dementia were more likely to have experienced 1 or more ED visits in the previous year >> Mean numbers of comorbidities were similar for both groups, though patients with dementia were more likely to have fluid and electrolyte disorders, additional neurodegenerative disorders and depression, but less likely to have cancer or be obese. >> Reasons for ED admission: patients with dementia presented higher rates of orthopedic trauma and renal procedures disorders, but lower rates of cardiac disorders and digestive system disorders. >> Length of stay was longer for patients with dementia, ED revisits were more frequent, and mortality was higher	High
24. Williamson LE35, 2023	Retrospective, observational study ED of public hospitals (United Kingdom)	74486/74486	Examine individual and service-level factors associated with ED visits by people with dementia in the last year of life	Not directly evaluated	-	>>In the last year of life, 82.6% (*n* = 61,491) of patients with dementia visited the ED, 53.2% (*n* = 39,596) attending at least twice. >> For 58.1% (*n* = 89,776) of all visits in the last year of life, the ED outcome was hospital admission. For 0.5% (*n* = 840), the ED outcome was death in the department. >>The proportion of visits that were in the month before death was 23.9% (*n* = 36,849), and for 66.7% (*n* = 24,569) of these, the ED outcome was hospital admission. >> Reasons for ED admission: mostly respiratory conditions and urological conditions, including cystitis >> Factors associated with higher end-of-life ED visits included: South Asian ethnicity (IRR 1.07, 95% CI 1.02–1.13,*p*P < 0.01), diagnosis of vascular dementia (IRR 1.14, 95% CI 1.13–1.16, *p*P < 0.01) or unspecified dementia (IRR 1.12, 95% CI 1.10–1.14, *p* < 0.01), underlying cause of death as chronic respiratory (IRR 1.33, 95% CI 1.28 1.38, *p* < 0.01), cardiovascular (IRR 1.17, 95% CI 1.14–1.20, *p* < 0.01), or cerebrovascular disease (IRR 1.14, 95% CI 1.11–1.18, *p* < 0.01), other underlying causes of death (IRR 1.22, 95% CI 1.19–1.25, *p* < 0.01), being a resident in an urban settlement (IRR 1.06, 95% CI 1.04–1.08, *p* < 0.01) and selected regions >> There was a negative dose–response association between ED visits and increasing socioeconomic position (IRR 0.92, 95% CI 0.90–0.94, *p* < 0.01). Being a resident in a local authority with more nursing home beds was statistically significantly associated with fewer ED visits (IRR 0.87, 95% CI 0.80–0.95, *p* < 0.01)	Medium

ED = emergency department; PC = palliative care; WOE D = Weight of Evidence D.15

^a^Secondary analysis of Kruhlak.27

### Outcomes and measures

Identification of patient’s characteristics, including reason for admission, treatment received, and destination were the most frequent outcomes (Amado-Tineo et al. 2020a; Chen et al. 2017; De Matteis et al. 2022; Glajchen et al. 2011; Hanson et al. 2019; Hunt et al. 2018b; Kruhlak et al. 2021; Lamantia et al. 2016; Leniz et al. 2021; Nordt et al. 2023; Reeves et al. 2023; Rosenwax et al. 2015; Straeuli et al. 2022; Willert et al. 2021; Williamson et al. 2023; Wong et al 2017), followed by frequency of ED visits (Afonso-Argilés et al. 2020; Antunes et al. 2021; Chen et al. 2017; Fürst et al. 2022; Hill et al. 2022; Hunt et al. 2018b; Lamantia et al. 2016; Leniz et al. 2021; Nordt et al. 2023; Reeves et al. 2023; Straeuli et al. 2022; Williamson et al. 2023). Factors associated with ED use were assessed in 5 studies (Afonso-Argilés et al. 2020; Hunt et al. 2018b; Rosenwax et al. 2015; Sleeman et al. 2018; Williamson et al. 2023).

Palliative care needs of patients attending the ED, including patients with dementia, were evaluated in 8 studies (Amado-Tineo et al. 2020a, 2020b; Glajchen et al. 2011; Kirkland et al. 2022a; Kruhlak et al. 2021; Leniz et al. 2021; Straeuli et al. 2022; Wong et al 2017), using different tools, namely GSF-PIG1 (Straeuli et al. 2022; Wong et al 2017), P-cares (Kirkland et al. 2022a; Kruhlak et al. 2021), BriefPal (Glajchen et al. 2011), NECPAL (Amado-Tineo et al. 2020a), SPICT-ESTM (Amado-Tineo et al. 2020ba), and Palliative Care Quality and Outcomes Framework (Leniz et al. 2021). Access to palliative care was evaluated in 7 studies (Amado-Tineo et al. 2020a; Fürst et al. 2022; Kirkland et al. 2022a; Kruhlak et al. 2021; Leniz et al. 2021; Rosenwax et al. 2015; Willert et al. 2021).

The 3 clinical trials included evaluated the feasibility of BriefPal as a screening tool (Glajchen et al. 2011), the development of a best practice model for patients with dementia (Fürst et al. 2022), and a care transition intervention (Shah et al. 2022).

### ED use and reasons for admission

Dementia represented 1 of the most frequent non-oncological primary diagnoses for patients admitted to the ED with palliative care needs (Glajchen et al. 2011; Kruhlak et al. 2021; Nordt et al. 2023; Straeuli et al. 2022). Infections (Chen et al. 2017; De Matteis et al. 2022; Hanson et al. 2019; Hunt et al. 2018b; Lamantia et al. 2016; Williamson et al. 2023), mostly respiratory or urinary, and neuropsychiatric symptoms (De Matteis et al. 2022; Hanson et al. 2019) were the most prevalent reasons for admission.

Emergency use by patients with dementia were consistently high (Afonso-Argilés et al. 2020; Antunes et al. 2021; Chen et al. 2017; Fürst et al. 2022; Hill et al. 2022; Hunt et al. 2018b; Lamantia et al. 2016; Leniz et al. 2021; Nordt et al. 2023; Reeves et al. 2023; Rosenwax et al. 2015; Williamson et al. 2023), mostly at the end of life (Chen et al. 2017; Hunt et al. 2018b; Leniz et al. 2021; Rosenwax et al. 2015; Williamson et al. 2023) and often with more than 1 visit in the last year of life (Antunes et al. 2021; Nordt et al. 2023; Rosenwax et al. 2015; Williamson et al. 2023). Analysis of factors associated with ED use suggested that being male (Rosenwax et al. 2015; Sleeman et al. 2018), younger (Rosenwax et al. 2015), and having depression (Sleeman et al. 2018), pain (Hunt et al. 2018b), polypharmacy, falls, weight loss, caregiver burden (Afonso-Argilés et al. 2020), type of dementia (Rosenwax et al. 2015; Williamson et al. 2023), and place of residency (Rosenwax et al. 2015; Williamson et al. 2023) might be associated with increased ED use. In contrast, access to community palliative care was associated with reduced use of ED (Straeuli et al. 2022).

### Palliative care needs and access to palliative care

Prevalence of palliative care needs of patients with dementia assisted at the ED ranged from 0.6% (Straeuli et al. 2022) to 7.9% (Glajchen et al. 2011). When comparing to patients with other comorbidities also assisted at the ED, prevalence of palliative care needs of patients with dementia was non-inferior to that of cancer patients (Kirkland et al. 2022a). Considering total dementia patients attending the ED, palliative care needs ranged from 18.3% (Glajchen et al. 2011) to 33.6% (Leniz et al. 2021). Access to palliative care was low in general (Amado-Tineo et al. 2020a; Kirkland et al. 2022a; Kruhlak et al. 2021; Leniz et al. 2021; Rosenwax et al. 2015; Willert et al. 2021), only 3 studies presented rates pertaining to patients with dementia, ranging from 6.0% (Rosenwax et al. 2015) to 42% (Willert et al. 2021). The latter study considered patients with dementia associated with cancer, inferior to the 76% of patients who only had cancer.

Studies included presented a WOE D quality score of medium (Afonso-Argilés et al. 2020; Amado-Tineo et al. 2020b; Glajchen et al. 2011; Hunt et al. 2018b; Kruhlak et al. 2021; Lamantia et al. 2016; Leniz et al. 2021; Nordt et al. 2023; Rosenwax et al. 2015; Sleeman et al. 2018; Straeuli et al. 2022; Willert et al. 2021; Williamson et al. 2023) or high (Afonso-Argilés et al. 2020; Amado-Tineo et al. 2020a; Antunes et al. 2021; Chen et al. 2017; De Matteis et al. 2022; Fürst et al. 2022; Hanson et al. 2019; Hill et al. 2022; Kirkland et al. 2022a; Reeves et al. 2023; Rosenwax et al. 2015; Shah et al. 2022).

## Discussion

To our knowledge, this is the first review directly addressing palliative care needs of patients with dementia at the ED at the end of life. Our findings reveal that the evidence is scarce, limited to 24 studies. Patients with dementia in the last year of life frequently resort to the ED, representing 1 of the main diagnosis of patients attending this service. Moreover, patients with dementia often seem to present to the ED near the end of life, frequently more than once in their last year of life. Only 8 studies directly addressed palliative care needs and a plethora of measurement tools were used, suggesting significant rates of palliative care needs among patients with dementia and in comparison, to other oncological or non-oncological conditions. Some of the main reasons of admission to the ED are infections and neuropsychiatric symptoms. Although access to community palliative care seems to reduce ED use, evidence to support this remains scarce.

Previous studies estimate that 32–35% of patients attending the ED present unmet palliative care needs (Kirkland et al. 2022b), presenting a wide range in prevalence, from 0.6% (Straeuli et al. 2022) to 7.9% (Glajchen et al. 2011), and these needs are probably non-inferior to the ones present in cancer patients (Kirkland et al. 2022a). These results are in accordance with studies describing similar levels of distressing symptoms and palliative care needs in patients with dementia and patients with cancer and other chronic diseases, outside the ED setting (Chaudhry et al. 2013; Eisenmann et al. 2020; Moens et al. 2014; Sampson et al. 2018).

ED use is usually considered a low-quality indicator of end-of-life care, including,patients with dementia, especially in the last 90 days of life (Gozalo et al. 2011; Leniz et al. 2019). In fact, ED attendance of patients with dementia has been associated with an increased risk of delirium, falls, cognitive and functional decline, readmission, and death (Godard-Sebillotte et al. 2019; Travers et al. 2014; Volicer and Simard 2015). Evidence shows that significant avoidable ED use occurs (Lamba et al. 2012; Nuñez et al. 2006). This is in line with our scoping review, given that this population presented high rates of ED use (Afonso-Argilés et al. 2020; Antunes et al. 2021; Fürst et al. 2022; Hill et al. 2022; Hunt et al. 2018b; Lamantia et al. 2016; Leniz et al. 2021; Nordt et al. 2023; Reeves et al. 2023; Rosenwax et al. 2015; Williamson et al. 2023), specially at the end of life (Hunt et al. 2018b; Leniz et al. 2021; Rosenwax et al. 2015; Williamson et al. 2023) and frequently more than once in the last year of life (Antunes et al. 2021; Nordt et al. 2023; Rosenwax et al. 2015; Williamson et al. 2023).

Frequent ED resource suggests frequent unmet needs, including palliative care needs. None of the studies included in this review specifically addressed which palliative care needs are more commonly present. Reasons to resorting to the ED may represent some of these needs and in the present review infections (De Matteis et al. 2022; Hanson et al. 2019; Hunt et al. 2018b; Lamantia et al. 2016; Williamson et al. 2023) and dominated neuropsychiatric symptoms (De Matteis et al. 2022; Hanson et al. 2019). Other reasons of resorting to the ED have also been proposed (Vasquez et al. 2019), such as reduced oral intake and inability to tolerate medication. Williamson et al. (2021) previously identified several individual, clinical, and environmental factors that can influence ED attendance by patients with dementia at the end of life, including palliative care that was associated with reduced ED use. In our study, depression (Sleeman et al. 2018), pain (Hunt et al. 2018b), polypharmacy, falls, weight loss, and caregiver burden (Afonso-Argilés et al. 2020) were identified as factors that can be associated with ED use when addressing palliative care needs.

This scoping review confirms that palliative care needs of patients with dementia attending the ED are frequent and access to palliative care remains scarce (Amado-Tineo et al. 2020a; Fürst et al. 2022; Kirkland et al. 2022a; Kruhlak et al. 2021; Leniz et al. 2021; Rosenwax et al. 2015; Willert et al. 2021). Evidence shows that the main barriers contributing to the latter include time constrains, lack of privacy and other space limitations, staff with low confidence in palliative care skills, and difficulties in relationship building with patients and families. Barriers related to limited community service provision and to particular aspects of patients with dementia that complicate palliative care recognition such as cognitive changes, communication difficulties, and the pattern of slow incremental decline have also been identified (Jamieson et al. 2016; Jurgens et al. 2012; Lillyman and Bruce 2016; Mataqi and Aslanpour 2020; Ryan et al. 2012; Verhoef et al. 2020). Additionally, health-care professionals tend to underestimate the potential role of palliative care for life-limiting conditions other than cancer (Shearer et al. 2014), and patients with life limiting conditions are frequently triaged as low priority patients (Hjermstad et al. 2013) at the ED, and can consume resources like over investigations and inappropriate treatment that fail to address their needs and goals of care (Lawson et al. 2008).

Considering that ED use by patients with dementia with palliative care needs is frequent, some authors have proposed to assume ED use as an opportunity to identify gaps in care, offer timely interventions, initiate end-of-life discussions, and refer to palliative care services (Elsayem et al. 2016; George et al. 2015; Grudzen et al. 2016, 2011; Ouchi et al. 2017, 2014). Lamba et al. (2013) suggest training of emergency staff in palliative care principles encouraging them as primary providers to seek the multi-professional palliative care service as means to improve access to palliative care. Our results are in line with these views (Glajchen et al. 2011; Hanson et al. 2019; Shah et al. 2022). The BriefPal project (Glajchen et al. 2011) showed feasibility of successful screening of palliative care needs and the value of staff education, reporting patient symptom reduction and improvement in referral to palliative care services. Hanson LC et al. (2019) conducted a trial of best-practice model of specialty palliative care triggered by ED use, and results show that the intervention group presented more elements of palliative care needs addressed; more frequent assessment and treatment of physical, neuropsychiatric, and spiritual symptoms; and increased communication and decision-making concerning end-of-life issues. The Shah et al. (2022) trial reported that a care transition intervention can improve transition of patients with dementia, specially of those with depression, reducing ED revisits, and increase odds of obtaining follow-up.

### Strengths and limitations

This scoping review complied with an established systematic method and examined the available evidence on experience and needs of a particularly frail subgroup of patients, in the challenging setting of ED. Our search included studies from a diverse range of countries and hence presents wide-ranging relevance. However, there are some limitations. Search was limited regarding number of databases, and no hand search of key journals was conducted, besides those based on references from previous reviews. We only considered English-, Portuguese-, and Spanish-language publications. The main focus was in studies addressing palliative care needs directly with specific tools but studies with indirect assessment of palliative care needs were also considered. Studies included in this review presented very diverse methodologies and frequently samples were not exclusively of dementia patients, limiting analysis of the results.

## Conclusions

Current evidence indicates that patients with dementia frequently resource to the ED in their last year of life with unmet palliative care needs. Scarce access to palliative care seems to pose an even greater challenge to care for these patients in the ED setting. Results from palliative care intervention studies (Elsayem et al. 2016; George et al. 2015; Glajchen et al. 2011; Grudzen et al. 2016, 2011; Hanson et al. 2019; Lamba 2009; Ouchi et al. 2017, 2014; Shah et al. 2022) at the ED are encouraging and suggest that ED can and probably should be seen as an opportunity to attend to palliative care needs and refer to palliative care services.

Future research regarding models of identification of palliative care needs and development of best practice approach along with palliative care principles at the ED might shed light in improving the response to palliative care needs of patients with dementia. Referral to palliative care services, advance discussion of goals of care and reinforcement of community palliative care services seems to be key to guarantee better access to palliative care.

## Supporting information

Vieira Silva et al. supplementary material Vieira Silva et al. supplementary material

## Data Availability

Further information about the search strategies and other supplemental material are available from the corresponding author on reasonable request.

## Supplementary material

The supplementary material for this article can be found at https://doi.org/10.1017/S1478951524001627.
