# Supplementary material for: Emergency department use and responsiveness to the palliative care needs of patients with dementia at the end of life: A scoping review
Source: Palliat Support Care. 2025 Jan 27;23:e51. doi: 10.1017/S1478951524001627 (PMC13166383; doi:10.1017/S1478951524001627)
Supplement: Vieira Silva et al. supplementary material [file S1478951524001627sup001.pdf]

Suplemental table 1. Search terms and strategy Medline (Pubmed)

Term 1 Dementia, Delirium, Wernick Encephalopaty, Amnestic Disorders, Cognitive Disorders, Alzheimer, Lewy Body, Cerebrovascular, Organic Brain disease, Organic Brain syndrome, Normal pressure hydrocefalus, Shunt, Benign senescent forgetfulness, Cerebral Deterioration, Cerebral Insufficient, Korsakoff, Creutzfeldt-Jakob, Huntington

Term 2 Emergency department, emergency room, emergency medicine, emergency care, acute care

Term 3 Palliative care, supportive care, end-of-life care, end of life, hospice.

Complete phrase (dementia OR delirium OR wernick encephalopaty OR amnestic disorder OR cognitive disorder OR alzheimer OR lewy body OR cerebrovascular OR organic brain disease OR organic brain syndrome OR normal pressure hydrocefalus OR benign senescent forgetfulness OR cerebral deterioration OR cerebral insufficient OR Korsakoff OR creutzfeldt-jakob OR huntington) AND (emergency department OR emergency room OR emergency medicine OR emergency care OR acute care) AND (palliative care OR supportive care OR end-of-life care OR end of life OR hospice)
